# Agentic Self-regulation of Capoeira Athletes of Different Sports Qualifications

**DOI:** 10.11621/pir.2024.0306

**Published:** 2024-09-15

**Authors:** Ilya A. Molodozhnikov, Alexander Sh. Guseinov

**Affiliations:** a *Kuban State University of Physical Culture, Sports and Tourism, Krasnodar, Russia*; b *Kuban State University, Krasnodar, Russia*

**Keywords:** mental self-regulation, agentic self-regulation, escapism, constructive coping, personal maturity, personality harmony

## Abstract

**Background.:**

In the present study, an attempt was made to address self-regulation at the agentic (higher) level, which contributes a person’s success not only in sports, but also in the organization of their own life. A group of capoeiristas was chosen as the sample.

**Objective.:**

To identify the features of agentic self-regulation in athletes engaged in capoeira with different levels of sports qualification.

**Design.:**

202 capoeira athletes, aged M= 29.6 years, SD=6.6, were interviewed. The sample was divided into three subgroups based on different sport types and comparisons were made on the scales of the following techniques: *style of self-regulation of behavior*, strategies for coping with stressful situations, *action control*, *personality protest activity*, *psychosocial maturity*, *personality harmony*. ANOVA analysis in IBM SPSS Statistics 26 program was used for these comparisons.

**Results.:**

The *Masters* subgroup outperformed the lower grade subgroups on the parameters: *action control in planning*, *assertiveness*, *personal self-regulation*, and *psychological defense* (p ≤ .05). The *Master Candidates* group had a significantly lower level of escapism (p ≤ .05), an indicator of destructive agentic activity, compared to the r*ated athletes* group. *Master Candidates* outperformed *Masters* in the coping strategy of caution (p ≤ .05) and outperformed *rated athletes* on personality harmony scales, including satisfaction with life, relationships with people, life self-determination, and life self-actualization (p ≤ .05). On the scales of conscious self-regulation, no significant differences were found between the three subgroups (p > .1).

**Conclusion.:**

As capoeiristas advance in sportsmanship they enrich their regulatory experience through the development of agentic qualities such as constructive coping strategies, personal maturity and personal harmony, while reducing the manifestation of destructive activity, such as escapism.

## Introduction

When considering the variety of studies concerning sport, there exists a diverse range of attitudes exhibited by researchers in relation to the issue of self-regulation. Several authors study available regulatory skills (Locke & Latham, 2013), focusing on specific aspects of self-regulation: monitoring of the current state (Balk & Englert, 2020), self-control (Baumeister, Vohs & Tice, 2007), volitional regulation (Shliapnikov & Ivannikov, 2021), coping behavior (Cosma et al., 2020), all of are certainly useful from an applied perspective. Other researchers have sought to understand the holistic personality of the athlete, identifying finding ways to reveal the psychological resources necessary for achieving and realizing high sportsmanship (Gorskaia, 2011; Vallerand, 2007; Ryan & Deci, 2017, et al.). We believe that such studies are possible when considering concepts such as self-determination, personal maturity, activity, and so on, which reflect the personal level of self-regulation and allow us to view self-regulation as the basis of agentic manifestation of a person.

### Main parameters and mechanisms of agentic self-regulation in sports activity

The most well-known concept in Russian psychology is the concept of conscious self-regulation developed by Konopkin - Morosanova (Konopkin, 2004). The legitimacy of such frequent reference to this concept within the framework of sports psychology is explained by the positive influence of conscious self-regulation on the achievement of goals in sport (Morosanova & Bondarenko, 2016; Guseinov, 2012; Gorskaia, 2011) and the overcoming of competitive stress (Belozerova, Bragina, Semikasheva & Silakova, 2021). The interaction of components of conscious self-regulation with forms of protest behavior was studied by Guseinov (2013). As a result of the research, it was found that the constructive form of protest — emancipation can enhance the athlete’s self-regulation and promote stability in results, while destructive forms of protest create a disharmonious athlete profile and hinder goal achievement. In general, conscious self-regulation is an important agentic resource for self-development and goal attainment. At the same time, as Morosanova points out, high rates of self-regulation can be observed in athletes with lower performance efficiency (Morosanova, 1991). This fact shows that conscious self-regulation, in some cases, is insufficient for goal achievement and requires the exploration of other psychological bases, such as volitional regulation.

At present, the dominant view of will is conceptualized as a control over action, which encompasses all processes that mediate intention (Kuhl,1994). In Leontiev, control over action is an indicator of personal potential as the potential for self-regulation (Leontiev, Ovchinnikova, Rasskazova & Fam, 2022). Athletes have a more effective type of volitional regulation and its evaluation than people who do not practice sports, as well as a better formed value-sense sphere, self-control (Shlyapnikov & Ivannikov, 2021). In basketball, players who are oriented toward action dunk the ball into the basket more forcefully and more often, while those oriented toward the state pass to a partner (Sahre, 1991). In contrast, weight lifting, which emphasizes the concentration of attention, state-oriented athletes are more successful. Memmert and Roth (2007) found that state-orientation correlates with a higher frequency of athletes listening to their coach’s advice, which has a positive effect on athletic performance.

Another regulator of athletes’ activity is coping behavior, which consists of either adapting to the requirements of a given stressful situation, or otherwise avoid the stressful situation entirely (Nartova-Bochaver, 1997). Elite athletes more often use problem-oriented strategies, search for social support, planning, and visualization, which distinguishes them as reflected in their performance, demonstrating the effectiveness of these strategies (Berilova & Raspopova, 2022; Nicholls, Polman, Levy, Taylor & Cobley, 2007). Recently, coping behavior is considered a manifestation of agentic behavior and the result of human maturity, self-awareness, an established worldview, and harmony (Kriukova, 2008; Guseinov, 2012).

The concept of self-reflection, as an arbitrary turning of human consciousness to oneself, has a regulatory potential. Athletes who have developed reflective skills have a tendency to seek performance improvement on their own and require less help from the coach as week as demonstrating a higher capacity for analyzing their own technical mistakes (Loviagina, 2020). Jonker, Elferink-Gemser & Visscher (2010) found that self-reflection, together with talent, allows an athlete to acquire attributes necessary for a particular sport and to reach an elite level of sportsmanship. Self-reflection appears in both deep and superficial forms. Deep reflection is associated with individuals possessing personal maturity, an established set of life goals, and moral values (Guseinov & Shipovskaia, 2019); superficial reflection, on the other hand, leads to extremely simplified forms of self-analysis and self-management.

The analysis of the presented concepts shows that researchers refer to personal maturity, which mediates the subject’s behavior, conscious self-regulation, and reflection. According to Sergienko (2011), personal maturity is the result of the coordinated work of the personality as a carrier of the inner world of the subject in achieving goals and selecting resources. In the subject-existence methodology, self-regulation and personality development are associated with of the development of personal meanings, including the creation of a meaning for one’s life, as well as the possibility of resolving the contradictions arising from the e process of existence (Riabikina, 2008). By mastering new existential perspectives, individuals have a tendency to become more mature and achieve a more authentic life, as demonstrated by athletes who demonstrate subjective existential outlooks when transitioning to alternate teams, taking on new roles, or requalification from an athlete to a coach. Although Pechersky’s (2024) research has shown that despite the fact that personal maturity is a resource that contributes to sports achievement, there remains an ambiguous correlation between the formation of personal maturity, which promotes self-acceptance and adequate gender identity, and the capacity for socialization, self-control, and tolerance.

Abulkhanova-Slavskaya’s (1991) study, illustrated the concept of an individual’s *life path*, as fundamental to the actualization of a personality and its capacity to create and fully realize one’s own life. Such reflections are relevant in the context of sports. As the analysis of sport biographies illustrate, it’s not the randomness of athletes’ journey to international recognition, but rather a conscious strategy for achieving their goals. The planning of one’s own life, and the embrace of sport as emblematic of a unique set of life values, leads to a form of self-fulfillment perhaps only fully possible for mature personalities that possess a well-formed sense of these core personal values. In this sense, sport serves as a catalyst for personal potential and existential satisfaction. The study by Liashenko, Omelchenko, Gatsko & Gnutova (2020) showed that in the hierarchy of values found in elite athletes, *spiritual satisfaction* and *achievement* are prized above that of *material status* which falls in third place.

The degree of success achieved in a sports career and its longevity is influenced by the extent to which a person is found to be harmonious with themselves and their environment. According to Motkov (2020), a harmonious personality is a person with an optimal organization of his personality, which is characterized by a predominantly positive attitude with people and the desire for self-development. At the heart of a harmonious personality lies a flexibility of behavior, manifested in the ability to change goals and strategies for achieving them depending on external conditions, as well as the ability to coordinate opposite requirements (uniformity and diversity, stability and change, and so on) (Leontiev, & Osin, 2014). Harmony can be achieved according to the development of a style of behavior, which Gosudarev (1989) referred to as *sports rationalism*. Athletes with this style of behavior are characterized by a realistic attitude to life, an ability to predict the development of situations, and a capacity for analyzing and organizing the conditions necessary for success. Athletes who exhibit sport rationalism achieve maximum results with optimal mobilization of efforts. Athletes with disharmonious styles (sports giftedness and sports obsession) are characterized by either minimal effort to achieve results or excessive effort and perfectionism (Gosudarev, 1989).

However, sport can be both a sphere of human self-fulfillment and cause various kinds of disharmonies. In particular, sports disharmonies are connected with the problems of premature professionalization. Thus, for adolescents and individuals in young adulthood, the consequence of early professionalization can cause a narrowing of a rounded self-concept, reduced self-esteem, egoism, conflict toward collective and individual values, and anxiety (Gorskaya, 2008). Sports psychology also studies *protest* characteristics of personality including *opposition*, *nihilism*, and *escapism* all of which, when combined with negative personality traits, can form a disharmonious regulatory style that hinders the growth of sportsmanship (Guseinov, 2012).

Thus, in modern sports the research of mental self-regulation and its agentic resources is increasingly being emphasized. This can be exemplified by martial arts in particular, with their philosophy and principles of spiritual development, and harmonious personal development. As studies have shown, judo, characterized by the absence of blows in contact, contributes to the formation of a broad set of important psychological qualities, leading to well-adjusted emotional regulation, psychological stability, self-confidence, attention, optimism and other positive attributes (Silva, Dias, Corte-Real, & Fonseca, 2018). Additionally, judoka differ from representatives of team sports according to higher indicators of conscientiousness (Bojanic, Nedeljkovi, Sakan, Mitic, Milovanovi & Drid, 2019).

Our research focused on the national sport of capoeira, which, in addition to elements of combat, also incorporates acrobatics, music, dance, philosophy. These diverse components contribute to the harmonious and versatile development of a person. The everyday practice of capoeira is a non-contact fight, which allows men and women to compete on equal terms. One of our studies has shown that by integrating different forms of human creative activity, and emphasizing the beautiful fight as opposed to victory at any cost, capoeira contributes to the enrichment of regulatory experience, the development of emotional intelligence and personal harmony (Guseinov & Molodozhnikov, 2021).

Based on the idea of the integrality of the phenomenon of self-regulation and the specificity of the sport of capoeira, the following research objective was set: to identify the features of agentic self-regulation in athletes engaged in capoeira across varying levels of sports qualification.

### Hypothesis

It was hypothesized that capoeira athletes will develop self-regulation system through agentic qualities (constructive coping strategies, personal maturity, personal harmony) with the growth of sports qualification.

## Methods

### Participants

The study sample consisted of 202 Russian capoeira athletes (55.4% men and 45.6% women) of different levels of sportsmanship. The average age of the respondents was M = 29.6 years, SD = 6.6. The participants were athletes from different capoeira schools including, Real Capoeira, Russian Capoeira Center, Capoeira Camara, Cordao de ouro, Dende, Familia ginga e raca, Portao de ouro, ACMB. Additionally, city groupings included, Moscow, Krasnodar, St. Petersburg, Rostov-on-Don, Kazan, and Kirov. In order to determine the differences between athletes of different qualifications, the total study sample was divided into 3 subsamples, the characteristics of which are presented in *Table 1.*

**Table 1:** Characteristics of the research sample

Subgroup	Number of people	Belt	experience Training	in Participation competitions
Masters	48 people — 37 male,11 female	instructor, professor	>10 years	International level
Master candidates	60 people — 38 males, 22 females	monitor	Ages 5–9	All-Russian level
Rated athletes	94 people — 37 males, 57 females	graduado, minitor	Ages 1–4	City level

### Procedure

During the capoeira training camp period (November 2021), 60 athletes were tested on paper. The remaining 142 athletes during the period spanning 2021 to 2022 were surveyed online via Google-forms. To facilitate ease of completion, the online questionnaires were grouped into two blocks, each taking 20–25 minutes to complete. All respondents gave permission to participate in the survey and to publish the results. Study participants were given feedback on the test results and a brief psychological report. The data collection procedure complied with the ethical standards of the Russian Psychological Society.

### Materials

The following psychological tests were used in the study:

1. *The Behavioral Self-Regulation Style-98 (BSS-98) questionnaire* (Morosanova, 2001). It consists of 46 statements assessing the main regulatory processes and regulatory-personal properties. Answers were given on a 4-point scale, where “1” is correct and “4” is incorrect. (Cronbach’s α is .72).
2. *The Stress Coping Strategies (SACS) questionnaire* (Hobfoll, 1994, Vodopianova, 2013). It consists of 54 statements designed to identify preferred strategies for coping with difficult (stressful) situations. Answers were given on a 5-point scale, where “1” means no, not at all true, “5” means yes, absolutely true (Cronbach’s α is .47–.78).
3. *The Action Control Questionnaire* (Kuhl, 1994, Shapkin, 1997). This questionnaire was adapted for the Russian-speaking sample by Shapkin and is designed to determine individual dispositions *action orientation* or *state orientation*. Respondents are asked to choose one of two options (*a* or *b*), where 1 point is given for matching the key. (Cronbach’s α is .70–.74).
4. * Personal Protest Activity Questionnaire* (PAQ) (Guseinov, 2015). The parameters derived from this methodological scale reveals a meaning full typology of both constructive and destructive protest attitudes derived from certain conceptual values including, *emancipation*, *escapism*, *negativism*, *opposition*, and *nihilism*. Responses complete the questionnaire by specifying a range between”0” points — absolutely disagree to “4” points — absolutely agree (Cronbach’s α is .60–.81).
5. *Psychosocial Maturity Questionnaire* (PMQ) (Pashnev, 2010). This questionnaire consists of 50 statements and reveals the general level of psychosocial maturity of the personality, as well as its components (attributes) including, *self-determination*, *self-regulation*, *ego strength*, *self-actualization*, *socialization*, *cognitive motivation*, and *psychological protection*. The respondent chooses answers based on a response range as follows, “a” — very rarely, “b”(— sometimes, “c” — often, “d” — almost always.6. *Personality Harmony Questionnaire* (PHQ) (Motkov, 2020). This questionnaire consists of 109 questions that allows for the analysis necessary to evaluate the integral overall harmony of personality. Responses are given on a 5-point scale, where “1” indicates very little; “5” indicates very much. (Cronbach’s α is .87).

### Data Analyses

One-factor analysis of variance (ANOVA) in the IBM SPSS Statistics 26 program was used to compare the averages. The sports qualification of the athletes was used as a factor, and the parameters of the above mentioned techniques were used as dependent variables. To check the suitability of the data for variance analysis, the homogeneity of variance was tested using Levene’s test. After establishing significant differences through ANOVA, multiple comparisons between groups were performed a posteriori using Tukey’s HSD test.

## Results

As a result of ANOVA analysis, in which the scales of conscious self-regulation (BSS-98) were used as dependent variables, it was revealed that there were no reliable differences between subgroups of capoeira athletes of different qualifications (see *Table 2*). That is, capoeira athletes of different sports qualification have equally developed abilities to govern their own actions, to select significant conditions, to plan, to be independent, and to evaluate their own actions. At the tendency level, capoeiristas of different qualifications differ in regulatory flexibility (p = .08), which probably indicates the favorable influence of capoeira on the ability to quickly adapt to changing environmental conditions.

**Table 2:** Average values of capoeiristas of different sports qualifications in self-regulation technique

Scale name	Masters		Master candidates		Rated athletes	
	*M*	*SD*	*M*	*SD*	*M*	*SD*
Planning	5.8	2.0	6.0	1.9	5.5	2.3
Modeling	6.6	1.7	6.2	1.7	6.3	1.9
Programming	6.4	1.5	6.3	1.5	6.5	1.8
Performance evaluation	6.7	1.4	6.3	1.7	6.4	1.3
Flexibility	6.6	1.6	6.9	1.3	6.1	1.9
Independence	4.8	1.8	5.2	2.0	4.5	2.2
General level of self-regulation	32.1	4.8	31.9	4.9	30.8	5.9

Comparison of the mean values of the three groups of athletes on the *Action Control* measure showed that at least two groups of capoeiristas differed on the *Action Control in Planning* scale (F(2,80) = 3.034, p = .05) (see *Table 3*). Moreover, a posteriori comparison on this scale using Tukey’s HSD test showed that *masters* athletes differed from *rated athletes* (p = .04, 95% CI = [.05, 4.37]). Consequently, indicators reveal that *masters* athletes have a greater degree of task-oriented ability compared to those in lower qualification levels; they are less likely to experience anxiety about an incomplete intention. For example, in the case of an unsatisfactory result at a competition, highly skilled athletes will analyze the situation and set further goals, while less skilled athletes will have thoughts about failure.

**Table 3:** Average values of capoeiristas of different sport qualifications according to the methodology of action control

Scale name	Masters		Master candidates		Rated athletes	
	*M*	*SD*	*M*	*SD*	*M*	*SD*
AC in planning	8.2*	3.6	7.2	3.2	5.9*	2.7
AC in realization	6.1	2.8	5.7	2.4	5.4	2.4
AC on failure	8.5	2.2	8.4	2.5	9.3	1.8

Note: *p ≤ .05; AC - Controlling for action

Comparison of mean values using the “Stress Coping Strategies” technique showed that the subgroups differed on the parameter of *assertiveness* (F(2,104) = 4.790, p = .01) and *cautiousness in action* (F(2,104) = 3.806, p = .025) (see *Table 4*).

**Table 4:** Average values of capoeiristas of different sports qualifications according to the coping-strategy methodology

Scale name	Masters		Master candidates		Rated athletes	
	*M*	*SD*	*M*	*M*	*SD*	*M*
Assertiveness	21.6*	2.8	19.9	3.1	19.3*	3.1
Making contact	22.9	3.1	23.6	3.0	24.3	3.0
Psychosocial support	22.6	2.9	24.2	3.5	24.2	4.5
Caution	19.1*	2.8	21.0*	2.9	20.1	3.5
Impulsiveness	18.0	2.8	17.4	3.0	18.3	2.9
Avoidance	16.0	3.7	16.7	3.4	15.7	3.1
Manipulative actions	16.9	2.9	18.4	4.3	17.3	4.3
Antisocial behavior	15.7	4.2	15.4	4.1	14.3	3.9
Aggressive actions	16.3	4.5	16.3	4.3	15.6	4.5

Note: *p ≤ .05

A posteriori comparison using the Tukey HSD test showed that, on the *assertive actions* scale, *masters* differed from the level-outs (p = .012, 95% CI = [.42, 4.06]). That is, *masters* athletes differ from less experienced athletes in activity, confidence in behavior, independence from external evaluations, and the ability to defend their point of view in constructive ways.

Reliable differences were also found between *masters* and *master candidates* on the *cautious actions* scale (p = .019, 95% CI = [.27, 3.62]). Underestimated indicators in the group of *masters* indicate that elite athletes act more decisively, confidently, can take risks if the risk is justified, and athletes give their best at sporting events without “saving their strength”. For the other parameters of this methodology no reliable differences were found.

Comparison of mean values of the *psychosocial maturity* (Pashnev, 2010) technique showed that athlete groups differ across parameters that include, *personal self-regulation* (F(2,177) = 4.038, p = .019), *psychological defense* (F(2,177) = 3.248, p = .041). The Tukey HSD multiple comparisons test showed that the mean value of personality self-regulation differed between the *masters* and the *master candidate* groups (p = .014, 95% CI = [0.16, 1.77]). The *psychological defense* parameter also showed differences between the *masters* and *master candidate* groups (p = .034, 95% CI = [.03, .93]). The table below indicates the mean values of the three groups of athletes on the PPD scales (*Table 5*). It indicates that *masters* have more developed qualities associated with self-regulation, such as endurance, self-discipline, purposefulness, strategic thinking, coping with negative emotions, belief in themselves, orientation to vital, valuable deeds. The elevated level of psychological defense in *masters* in contrast with less qualified athletes indicates a refined ability to be consistent with their messaging, logical in their thinking, and demonstrate a capacity to admit one’s mistakes and laugh at oneself.

**Table 5:** Average values of capoeiristas of different sports qualifications according to the methodology of psychosocial maturity of personality

Scale name	Masters		Master candidates		Rated athletes	
	*M*	*SD*	*M*	*SD*	*M*	*SD*
Self-determination	8.6	1.4	8,3	1.7	8.5	1.7
Personal self-regulation	7.8*	2.1	6.8*	1.9	7.2	2.4
The power of the ego	3.9	1.3	3.9	1.1	3.6	1.4
Self-actualization	4,1	1.1	4.2	0.9	4.0	1.1
Socialization	6.8	1.7	6.6	1.5	6.1	2.2
Cognitive motivation	2.1	.7	2.2	.8	2.2	.8
Psychological defense	6.1*	.9	5.6*	1.2	5.7	1.4

Note: *p ≤ .05

A comparison of the mean values of the *personality harmony* technique indicates that capoeirista groups differ across parameters that include, *satisfaction with life and relationships with people* (Ud) (F(2, 96) = 5.340, p = .006), *life self-determination* (F(2, 96) = 4.812, p = .010), *life fulfillment* (F(2, 96) = 3.923, p = .023), and *positivity of self-esteem* (F(2, 96) = 3.472, p = .035). The results of the ANOVA are presented in *Table 6*. Tukey’s HSD multiple comparisons test indicate that the mean of the Ud scale was significantly different between the *masters’* candidate and *discharge* groups (p = .016, 95% CI = [.6, .66]). That is, elite athletes who achieve elevated results in sports are more satisfied with life. The situation is similar for the *life self-determination* assessment (p = .012, 95% CI = [.8, .73]) and *life self-realization* assessment (p = .037, 95% CI = [.2, .67]). That is, more skillful athletes are characterized by personal maturity, life skills, values, a tendency toward increased life planning, and a satisfaction with their successes and achievements. Despite the clear difference in the mean values between the groups according to the *positivity of self-esteem* parameter, no reliable differences were found using the Tukey HSD test.

**Table 6:** Average values of capoeiristas of different sports qualifications according to the methodology of personality harmony

Scale name	Masters		Master candidates		Rated athletes	
	M	SD	M	SD	M	SD
Vph	4.1	.6	4.1	.4	3.9	.4
Spiritual values	4.0	.3	4.0	.4	3.9	.3
Lifestyle	4.1	.4	4.1	.4	4.0	.4
Sr	3.7	.5	3.8	.4	3.6	.4
Constructive communication	3.9	.5	3.9	.4	3.8	.4
Self-harmonization of personality	3.8	.5	3.8	.5	3.7	.4
Mda	3.3	.4	3.7	.6	3.6	.5
Independence	4.1	.7	3.9	.5	3.7	.6
Ud	4.0	.5	4.0*	.5	3.6*	.5
Life self-determination	3.6	.6	3.7*	.6	3.3*	.6
Life fulfillment	3.7	.5	3.7*	.6	3.3*	.6
Positivity of self-esteem	4.1	.3	4.0	.4	3.8	.5
IHL	3.9	.4	3.9	.3	3.7	.3

Note: *p ≤ .05; Vph - Values of personal harmony; Sr - Self-regulation; Mda - Moderate power of desires and achievements; Ud - Satisfaction with life and relations with people; IHL - Integral index of harmony

A comparison of mean values on the *personality protest activity* technique indicated that at least two groups of respondents differed from each other on the *escapism* parameter (F(2, 80) = 3.495, p=.035)*.* The Tukey HSD multiple comparisons test indicated that the mean value on the *escapism* parameter differed significantly between the master candidate and discharge candidate groups (p = .031, 95% CI = [.3; 7.58]). The mean values for all scales of the PAL are shown in *Table 7*.

**Table 7:** Average values of capoeiristas of different sports qualifications according to the methodology of personality protest activity

Scale name	Masters		Master candidates		Rated athletes	
	M	SD	M	SD	M	SD
Negativism	20.5	6.0	20.1	6.0	23.4	8.7
Emancipation	18.9	3.1	19.9	1.9	20.0	2.4
Opposition	9.4	4.6	10.5	4.2	12.3	5.5
Anomie	6.3	3.7	5.7	3.4	6.8	4.8
Escapism	12.1	5.7	11.8*	3.7	15.7*	5.9

Note: *p ≤ .05

## Discussion

Our study showed that according to the *Behavioral Self-regulation Style-98* questionnaire (Morosanova, 2001), no significant differences were found among capoeiristas with varying levels of sports qualification. There were observed tendencies of differences among the subgroups regarding regulatory flexibility (p = .08). However, studies by other authors using the same technique have recorded contradictory results. According to the results of Lovyagina (2016), reliable differences were identified between elite athletes and those in mass categories regarding only one parameter of self-regulation: *evaluating the results of their own activity* In the study by Bosenko (2013), elevated differences were found across groups of boys and girls engaged in taekwondo and handball. In the work of Chub (2017), representatives of mass discharges surpass elite athletes in the *flexibility* and *evaluation of activity results* parameters. Pirozhkova (2013) correlates the characteristics of conscious self-regulation with the activity type according to team and individual sports. It can be assumed that the absence of differences in self-regulation style among athletes of varying levels may be attributed to the relatively low demands of capoeira. This sport is aimed at holistic human development and harmonious integration of all regulatory attributes; thus, we do not observe accentuations.

*Masters* have an elevated ability to be task-oriented when compared to *level-players*. Moreover, they are less likely to experience anxiety concerning efforts that remain incomplete. In their work Shlyapnikov and Ivannikov (2021) observe that the development of sportsmanship is associated with the growth of an athletes volitional qualities and their self-assessments. For example, research of basketball athletes has found that action-oriented players are more successful in sports due to c a capacity to make quick decisions, demonstrate an efficiency in response, and competently manage resources (Sahre, 1991). In martial arts, action-oriented athletes have been found to be better at maintaining confidence, anticipating opponent movements, and reacting quickly (Beckmann & Kazén, 1994). Thus, our findings on the effectiveness of action orientation in capoeira are consistent with studies conducted in other sports, indicating the universality of this trend.

The subgroup of *masters* surpasses *rated* athletes according to *coping-strategy assertiveness*. This means that *masters* are more active, confi dent, and independent. *Masters* have developed the ability to constructively defend their point of view, and they are less dependent on assessments from others. In the part of coping assertive actions our study is consistent with a study concerning fire-applied sport (Afanasieva, Ilina, & Svitlychna, 2023).

With increasing skill levels, it has been observed that capoeiristas’ escapism decreases in relation to destructive agentic activity levels, which is consistent with the Guseinov (2013) study conducted with a sample of athletes of different specialization. Escapism is considered a manifestation of the refusal to actively master the world, marked by a high level of social anxiety, non-acceptance of oneself, sensitivity to the opinion of others, weakness of will, dependency, and external locus of control. Probably, the decrease in escapism with the growth of sportsmanship is associated with the gradual involvement of an individual in sports and the mastery of various aspects of capoeira, which provides a structured vector for self-development.

*Masters* have developed qualities characterizers of personal maturity indicative of agentic self-regulation, attributes that include: these are endurance, self-discipline, purposefulness, strategic thinking, coping with negative emotions, belief in oneself, and an orientation to vital matters. This elevated level of psychological fortitude is reflected in an ability to act consistently, logically, with a capacity to recognize personal mistakes. *Masters* are characterized by personal maturity, life skills, values, a tendency to life planning, and a satisfaction with their successes.

The results obtained through this study confi rm the hypothesis that with the growth of capoeirista sportsmanship there is a development of agentic self-regulation. Moreover, this development occurs not in terms of structural components of self-regulation, but through the increasing agentic potential of the personality.

## Conclusion

The increasing level of sports qualification leads to a decrease in escapism, or escape from reality, which is an indicator of destructive agentic activity. With the growth of sportsmanship, capoeiristas enhance not merely their ability for conscious self-regulation, which determines the success of achieving current activity goals, but also the development of personal qualities that expand their regulatory experience, enabling them to achieve better sports results.

Sports qualification contributes to the mobilization of agentic resources associated with indicators of constructive coping, personal maturity and harmony. These components form a set of qualities that determine the agentic self-regulation of athletes. The conducted research brings clarity to the specificity of the regulatory experience of capoeira athletes and, presumably, similar sports with a creative and developmental orientation.

## Limitations

The comparison of mean values was based on one criterion only and did not take into account such criteria as age and gender. In the study, data collection was carried out using the test diagnostic method, which has its own disadvantages (agentic assessment, *blind* (automatic) errors, duration of performance, differences between live and online implementation, etc.). In the future, it is planned to conduct a similar study on athletes engaged in contact martial arts, so that it will be possible to compare the results.
